# Evaluating Cancer-Related Biomarkers Based on Pathological Images: A Systematic Review

**DOI:** 10.3389/fonc.2021.763527

**Published:** 2021-11-10

**Authors:** Xiaoliang Xie, Xulin Wang, Yuebin Liang, Jingya Yang, Yan Wu, Li Li, Xin Sun, Pingping Bing, Binsheng He, Geng Tian, Xiaoli Shi

**Affiliations:** ^1^ Department of Colorectal Surgery, General Hospital of Ningxia Medical University, Yinchuan, China; ^2^ College of Clinical Medicine, Ningxia Medical University, Yinchuan, China; ^3^ Department of Oncology Surgery, Central Hospital of Jia Mu Si City, Jia Mu Si, China; ^4^ Geneis Beijing Co., Ltd., Beijing, China; ^5^ Qingdao Geneis Institute of Big Data Mining and Precision Medicine, Qingdao, China; ^6^ School of Electrical and Information Engineering, Anhui University of Technology, Ma’anshan, China; ^7^ Beijing Shanghe Jiye Biotech Co., Ltd., Bejing, China; ^8^ Department of Medical Affairs, Central Hospital of Jia Mu Si City, Jia Mu Si, China; ^9^ Academician Workstation, Changsha Medical University, Changsha, China; ^10^ IBMC-BGI Center, T`he Cancer Hospital of the University of Chinese Academy of Sciences (Zhejiang Cancer Hospital), Institute of Basic Medicine and Cancer (IBMC), Chinese Academy of Sciences, Hangzhou, China

**Keywords:** histopathological image analysis, cancer biomarker, deep learning, color normalization, feature extraction

## Abstract

Many diseases are accompanied by changes in certain biochemical indicators called biomarkers in cells or tissues. A variety of biomarkers, including proteins, nucleic acids, antibodies, and peptides, have been identified. Tumor biomarkers have been widely used in cancer risk assessment, early screening, diagnosis, prognosis, treatment, and progression monitoring. For example, the number of circulating tumor cell (CTC) is a prognostic indicator of breast cancer overall survival, and tumor mutation burden (TMB) can be used to predict the efficacy of immune checkpoint inhibitors. Currently, clinical methods such as polymerase chain reaction (PCR) and next generation sequencing (NGS) are mainly adopted to evaluate these biomarkers, which are time-consuming and expansive. Pathological image analysis is an essential tool in medical research, disease diagnosis and treatment, functioning by extracting important physiological and pathological information or knowledge from medical images. Recently, deep learning-based analysis on pathological images and morphology to predict tumor biomarkers has attracted great attention from both medical image and machine learning communities, as this combination not only reduces the burden on pathologists but also saves high costs and time. Therefore, it is necessary to summarize the current process of processing pathological images and key steps and methods used in each process, including: (1) pre-processing of pathological images, (2) image segmentation, (3) feature extraction, and (4) feature model construction. This will help people choose better and more appropriate medical image processing methods when predicting tumor biomarkers.

## Introduction

Biomarkers are critical in cancer diagnosis, treatment, and prognosis. They can be used for patient’s evaluation in a variety of clinical settings, such as risk assessment, early diagnosis, drug effect evaluation, and prognosis prediction (1–3). With the development of immunology, molecular biology and genomics, studies of cancer biomarkers have attracted a lot of attention in recent years (4). Currently, biomarker identification usually employs technologies such as PCR, NGS and gene expression arrays (5). However, the data generated by these technologies need to be analyzed and interpreted manually. In addition, this kind of test usually costs a lot of money. For example, the test of tumor mutation burden (TMB) usually costs more than one thousand dollars. Thus, it will be of great value to develop a more intelligent and economical method in tumor biomarker identification (6).

Pathological image analysis is used to solve problems related to medical images which were applied in biomedical research and diagnosis. Its main objective is to extract clinically relevant physiological and pathological information or knowledge from images, and its main research direction is image segmentation, classification, and retrieval (7). With the rapid development and popularization of medical imaging technology, the amount of medical image data is growing rapidly. It will provide important and beneficial support for nursing and medical research to extract useful knowledge and information automatically from massive medical image data for clinical diagnosis and treatment (8). Recently, researchers have paid much attention to the analysis and study of tumor patients through pathological images and morphology (9). Mobadersany (10) proposed that the morphological characteristics of tumor tissue images could reflect the genetic and molecular characteristics and predict the degree of tumor deterioration, and the deep learning method could be used to integrate the morphological characteristics of tumor tissue images and genomics to predict the survival rate of glioma patients. Xu (11) proposed a method based on deep tissue network to automatically distinguish 10 tissue components in the colorectal full-scan tissue image. Yu (12) for the first time constructed the recurrence risk prediction model of LUAD and LUSC by automatically extracting morphological features from the full-scan histopathological images of lung cancer to provide prognostic information for patients. Vaidya (13) proposed to combine radiology and pathology to predict the risk of early lung cancer recurrence, with an accuracy rate of 70%. Wu (14) and others constructed a deep convolutional neural network framework to evaluate the risk of lung cancer recurrence and metastasis from histopathology images, with the area under the receiver operating characteristic (ROC) curve (AUC) in the test dataset of 0.79. Jain and Massoud explored a machine learning algorithm named Image2TMB to predict TMB from lung adenocarcinoma histopathological images. Its average precision was 0.89 and achieved predictive level of a panel of ~100 genes. Microsatellite instability (MSI) was another immunotherapy biomarker (15) which requires additional immunohistochemical or genetic analyses in clinical practice (16). Kather et al. developed a deep residual learning method that can predict MSI status directly from hematoxylin and eosin (H&E) stained histology slides (17). These findings suggest that inferring genomic features from histopathological images is possible and analyzing histopathological images is important for studying cancer treatments, mutated gene expression status, cancer prognosis and risk of recurrence.

**Figure 1:**
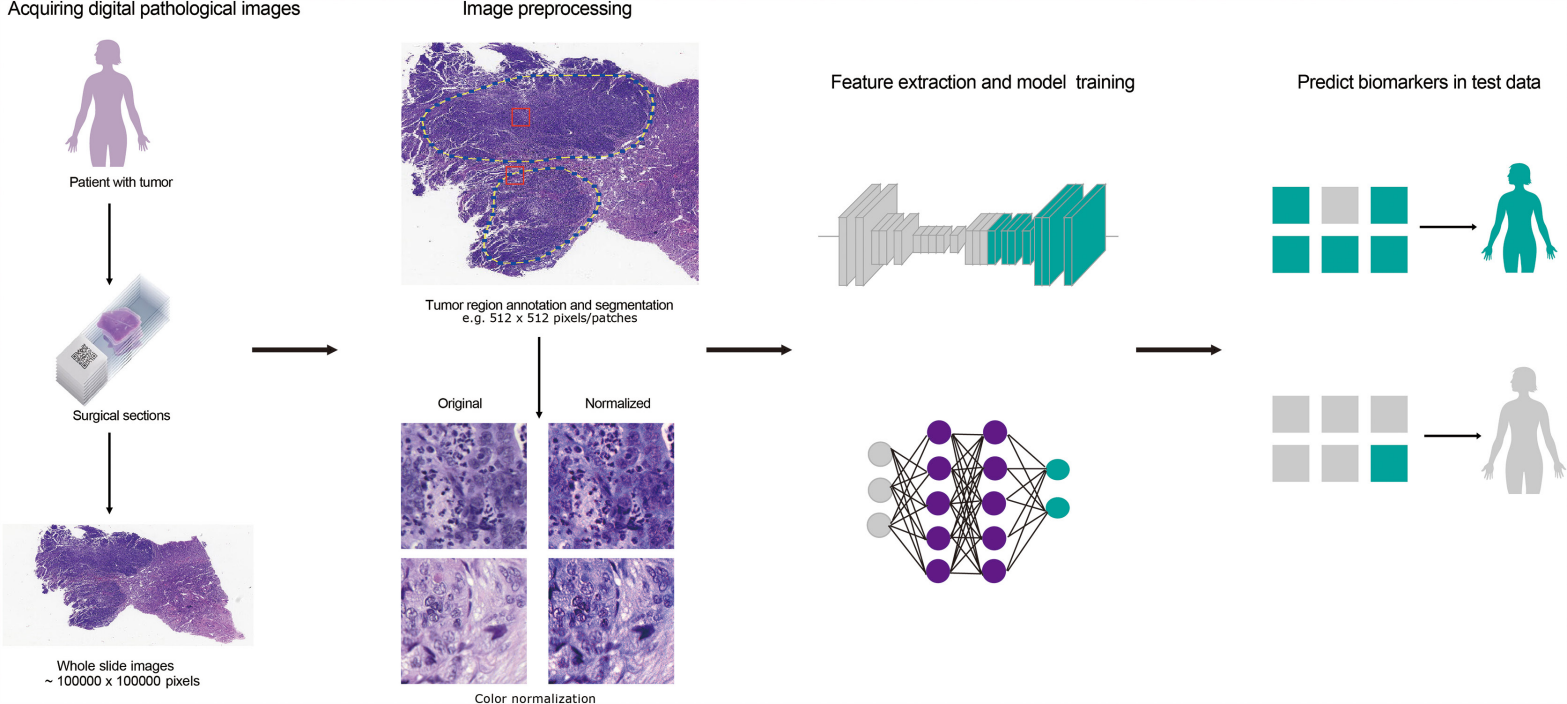
The flow chart for predicting cancer-related biomarkers based on digital pathological images. Firstly, H&E stained histology slides of patients were obtained and whole slide images (WSIs) was obtained after scanning. Secondly, tumor regions were annotated by pathologists or through CNN model. Then, the regions were segmented to patches and color-normalized. Thirdly, feature extraction and model training were carried out according to biomarker labels. Finally, biomarker prediction was implemented in test dataset.

However, full-scan histopathological images are highly complex, with large image size and about 2 GB of storage space after compression. It is a big challenge for hardware and image analysis algorithm to use computer to process image directly in this kind of high resolution and large size image. At the same time, the histopathological structure types in the images are disordered, and the histological morphology is very different, so it is difficult to describe with fixed features. All these factors bring great difficulty to the processing of full scan histopathological images. Based on the above problems, this paper summarizes the whole process and key steps of current pathological image processing, including image preprocessing, image segmentation, feature extraction and model construction, to help researchers choose more suitable medical image processing methods and predict biomarkers more accurately. We summarized the overall flow of pathological image processing in **Figure 1**.

## Image Preprocessing

The biggest obstacle to histopathological image analysis is the difference in image morphology due to high heterogeneity of the disease itself. At the same time, improper tissue treatment or staining during the slice preparation will result in morphological changes of cells and tissues, making it difficult to identify its original structure. In addition, the background noise and the lack of contrast caused by the different light source conditions were also important factors. Proper preprocessing method can correct images by eliminating irrelevant information, and filter out interference and noise, which can improve the detectability of target information and simplify the calculation to the maximum extent.

Common preprocessing methods such as using spatial filtering techniques to enhance the main structure in the image, image enhancement can improve the contrast between the region of interest and the background, and color normalization can reduce the effect of staining batches (18, 19). Among these, color normalization is the most commonly used image preprocessing methods for evaluating cancer-related biomarkers based on histopathological images.

### Color Normalization

In response to the problem of color change, Reinhard (20) and others proposed a method of color normalization, that is, in the lαβ color space, the mean and standard deviation of each channel in the image are compared with the target by a set of linear transformations. Then, match the mean and standard deviation. However, if multiple patches are used, the assumption of a unimodal distribution of pixels in each channel of the lαβ color space is not valid. Therefore, this may cause the background area to be mapped as a colored area and the foreground to be incorrectly mapped. As shown in **Table 1** below, some methods of color normalization were summarized.

**Table 1:** A summary of color normalization methods.

Authors	Methods	Characteristics	References
Magee	A method based on supervised pixel classification	Estimate the color of the coloring.	(21)
Macenko	A method based on singular value decomposition (SVD)	Direct estimation matrix.	(22)
Niethammer	An improved method based on singular value decomposition (SVD)	By expanding (22), a priori estimation of staining matrix is used to improve the stability of each staining.	(23)
Khan	Nonlinear mapping based on source image to target image	An improvement is proposed on the method of (21), using the representation method of color deconvolution.	(18)
Vahadane	A technique of dye separation and color normalization (SPCN)	It does a good job of maintaining the quality of biological structure and the number of stains.	(24)
Ramakrishnan	The improved SPCN	In the SPCN technology, some improvements are proposed for the occasional errors in estimating color bases, which lead to artifacts.	(25)

## Image Segmentation

Medical image segmentation is a complex and critical step in the field of medical image processing and analysis. The purpose of this process is to segment certain parts of the medical image with specific meaning, extract relevant features, and then provide reliable information for clinical diagnosis and pathological research. The two most common types of medical image segmentation are tissue segmentation and cell segmentation.

### Tissue Segmentation

Pathologists have identified that degree of structural differentiation of the tissue is one of the earliest prognostic factors for breast cancer patients. Cancer destroys the ability of the nucleus to communicate with each other and causes it to organize itself into structures such as tubules, thereby making the tubules lack of indicators of advanced malignant tumors. Tubules are usually round or oval in structure and consist of a lumen surrounded by a layer of epithelial cells. The main challenge of tubule segmentation is that it has a similar appearance to other structures, such as the tearing of adipose tissue formed during tissue preparation, and the outer layer of well-arranged epithelial cell with nuclei missing.

For glandular segmentation, most of the early attempts used hand-made features for segmentation. Wu (26) identified the initial seed region based on large cavity regions and extended the seed to the surrounding epithelial nuclear chain. Farjam (27) proposed using a variance filter to compute cluster texture features for segmentation. However, robust segmentation requires more domain knowledge, and calculating texture features only using the variance filter may not provide sufficient information for the local structure of the organization. Naik (28) used a Bayesian classifier to detect the lumen region, and then used the kernel-based level set to stop the curve and refine it. Although this method has been reported to work well in benign cases, it may fail in malignant cases with fairly complex glands. Nguyen (29) used color space analysis to group the nucleus, cytoplasm, and lumen, and increased the lumen area to achieve segmentation under constraints. Gunduz-Demir (30) represented each tissue component as a disc and connected nearby discs with an edge to construct a graph. They performed area growth on a cavity disc bounded by a line connected to the nuclear disc. Nosrati, Hamarneh (31) and Cohen (32) first divided the tissue area into different components, and then used a constrained level set algorithm to segment the glands. Sirinukunwattana (33) identified epithelial superpixels and used the epithelial region as the vertex of a polygon, which approximated the boundary of a gland. Most of the methods discussed above first distinguish tissue regions and then use region growth or level sets to segment glandular regions. Recently, a slightly different approach that first used background information to identify potential epithelial regions, and then used multi-resolution cell localization descriptors to identify connected epithelial cells to segment glands was proposed by Li (34).

### Cell Segmentation

The morphology of cells in histopathological images provides important information for the diagnosis and prognosis of cancer. Researchers at home and abroad have tried a variety of algorithms to solve the problem of cell segmentation in H&E images (34–36). The algorithms generally divided into two categories, one is to detect single cells accurately and the other is to segment cells. The algorithms in **Table 2** is commonly used to detect the appropriate seed point or contour of the nucleus.

**Table 2:** A summary of methods on segmentation after detection of individual cells.

Methods	Characteristics	References
Based on different voting rules	Simple and suitable for segmentation of most images	(11, 37–39)
Based on Laplace operator and gaussian filter	Accurately detect the edge of the cell	(40)
Based on H-minima transformation	Effectively restrain oversegmentation and reduce undersegmentation	(41)
Based on Morphologic manipulation	Could output an image by acting a structure element on the input image	(42, 43)
Based on back propagation with MRF	Good at dealing with the problems of image local volume and artifacts	(34)
Based on the active contour model	Could convert pixels to a distance field	(43)
Based on the level set	A numerical method based on the theory of geometric active contour model	(37, 44)

The other type detects the candidate area of the cell and then divides it into individual nuclei. The first step in morphological analysis of a cell is the segmentation of individual nuclei, which is usually performed manually in current clinical practice. However, due to the large volume of histopathological images and complex cell structures, manual examination is a time-consuming and labor-intensive task. It is necessary to study computerized methods to reduce the workload of pathologists and improve the analysis efficiency (45). Nuclear segmentation tasks still have some major challenges. First, different types of organs or cells are highly heterogeneous in appearance. Therefore, the method based on prior knowledge of geometric features cannot be directly applied to different images. Second, some other structures, such as the cytoplasm and cell matrix, may have similar characteristics to the nucleus, making it difficult to distinguish the nucleus from the background. Third, the cells are often stacked together. In order to find the exact location and boundary of each nucleus, it is usually necessary to perform the next step to separate the clustered or overlapped nuclei.

In view of the importance of nuclear distribution and morphology, the task of using computer algorithms for accurate nuclear segmentation provides a logical starting point for computer-aided tissue image analysis. The precise segmentation of the nucleus can not only perform deeper level feature extraction and classification in the nucleus, but also serve as a relatively simple distribution of basal cells and acellular cells. Many techniques have been applied to the task of nuclear segmentation, but in some cases they have only achieved partial success. For example, the intensity threshold method usually fails due to image noise and nucleus clustering. Label-based watershed segmentation requires accurate parameter selection, while the computational cost of active contours and deformable models is too high (24, 42, 46–50). Machine learning-based kernel segmentation methods are generally better at meeting these challenges because they can learn to recognize changes in nuclear morphology and staining patterns. More precisely, convolutional neural networks (CNNs) have recently demonstrated their latest performance in kernel segmentation (51, 52). Ciregan (53) applied deep CNN to the automatic detection of mitotic cells in breast cancer histological images. Using the original intensity of the test image, CNN provides a probability map where each pixel value is the probability of the mitotic cell centroid. Then using the disk to check the probability map for smoothing, and non-maximum suppression to get the final centroid detection. Xing (54) and others respectively learned three different CNN models corresponding to pathological images of brain tumors, pancreatic neuroendocrine tumors, and breast cancer, and applied them to automatic nuclear detection. Liu and Yang (55) did not use simple non-maximum suppression to refine the detection, but converted the detection problems of pancreatic neuroendocrine and lung cancer cell nuclear into optimization problems. Xing (47), Sirinukunwattana (51) and Song (55) have proposed some advanced techniques in nuclear detection and segmentation, which estimate the probability of nuclear and non-nuclear regions (both types) based on the learned nuclear phenomena graphs and rely on complex post-processing methods to obtain the final core shape and the separation between contacting nuclei. Song et al. used a graph partitioning method (55) and Xing et al. used a kernel mapping distance transformation, followed by H-minima thresholding and region growth (47). Although different methods have been developed for the problem of overlapping and clustering nuclei in many literatures, and have achieved varying degrees of success, this problem has not been completely solved, as there is a large amount of overlap contact specimens of nuclei.

In addition, a special type of nucleus, mitosis, has attracted much attention in the field of image analysis. Mainly because the mitotic index is used to evaluate the cell proliferation rate of cancer cells, it could predict the prognosis of invasive breast cancer well, but its evaluation process is extremely time-consuming (56). On the H&E image, mitosis has specific morphological features: dense nuclear staining, enlarged nuclei, less clear nuclear membrane, and burr-like edges. Researchers such as Belien (57) proposed image processing technology for semi-automatic segmentation of mitotic images in the 1990s. Due to the limitations of the image quality and machine learning algorithms at the time, the algorithm proposed by Belien et al. (57) required fourgen staining to display chromosomes, and the false positive rate is 19-42%. With the digitization of pathological images, two H&E tissue datasets of breast cancer have been published internationally, and pathological experts have annotated mitotic images in the images, which has greatly promoted the development of algorithms in mitotic image segmentation. Then, the International Conference on Pattern Recognition (ICPR) (58) held a competition for mitotic detection in breast cancer tissue images in 2012, providing different types of images, allowing participants to analyze classic images of H&E stained sections, and use 10 bands multispectral microscope images, which may be more discriminatory for detecting mitosis. Deep learning maximizing CNN significantly outperforms other manual feature-based methods and paves the way for future use of CNNs (53).

The biggest challenge for mitosis detection is that apoptosis, necrosis or squeezed nuclei and lymphocyte nuclei have similar morphology to mitosis, which is difficult for even experienced pathologists to identify. In addition, pathologists need to observe suspicious split images on multiple focal planes, while currently digital images are single focal plane imaging. Although some scanners can acquire multifocal plane images, their storage capacity is large and cannot be widely used. We expect that in the future, as storage costs decrease and new image compression technologies emerge, this limitation will be eliminated (59). Therefore, the automatic segmentation of mitotic images in H&E images at this stage is more challenging than general nuclear segmentation and is far from being applicable to pathological work.

## Model Construction

After the ideal segmentation results were obtained from the tissue segmentation and nuclear segmentation modules, the morphological features of histopathological images were extracted, and the correlation between the morphological features and biomarkers of the full-scan histopathological images was found and the feature model was established.

Beck et al. constructed a computer pathologist system to extract 6,642 dimensional features from H&E histopathological images of breast cancer (60). Some of the features are based on the existing knowledge system, such as the formation degree of counting glandular tube after automatic segmentation (61) and automatic grading (62), but most of the features go beyond the existing descriptive semantics of pathology. Computer-aided diagnosis is also based on the prognosis of characteristic models, modeling based on object characteristics, and then estimating the prognosis of model parameters. Tutac (63) proposed a semi-automatic grading system based on knowledge model for the first time, which automatically detected and measured the three components of histological grading, namely nucleus, adenotuine and mitosis, through semantic retrieval. The consistency of the scoring results of this model was higher than that of manual evaluation. Dalle (64) further improved the above work based on multi-resolution method and Gaussian model function, realized automatic histological classification, and the automatic classification results were highly consistent with the manual evaluation results.

**Figure 2:**
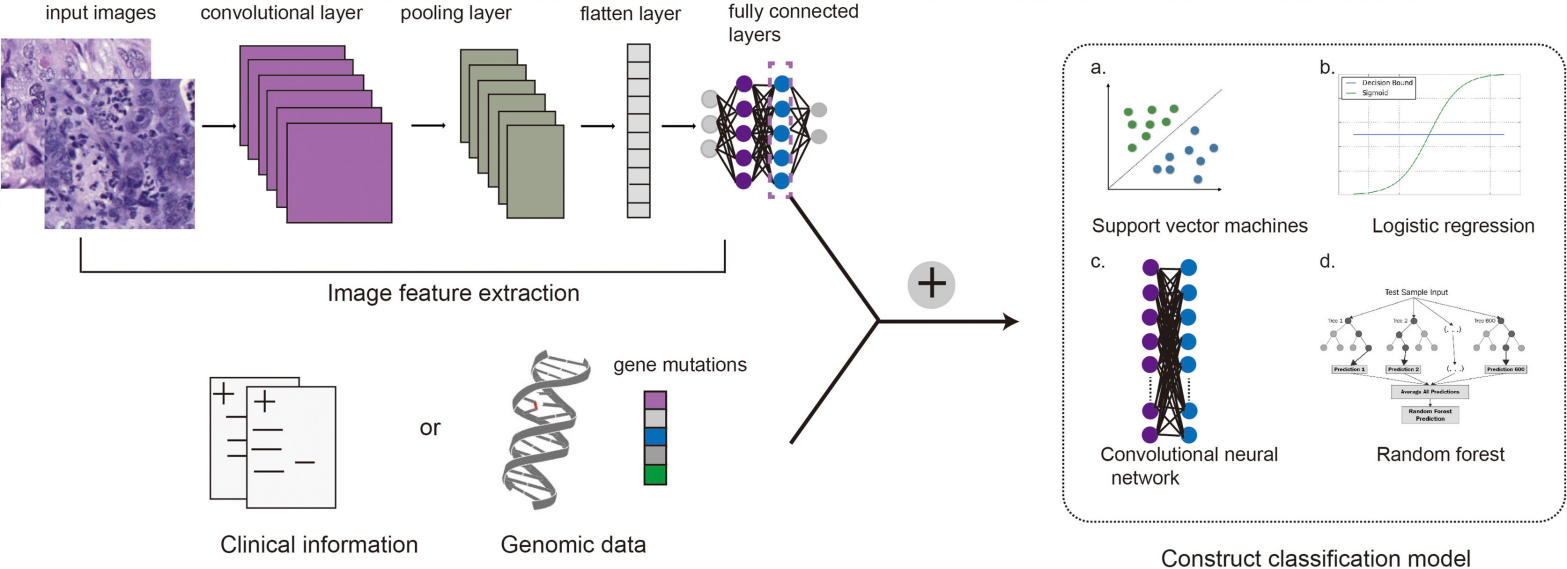
Main process of constructing a compound framework by combining pathological images with genomic data or clinical information. Convolutional neural networks are commonly used to extract image features, and then genomic features or clinical information are integrated into the full connection layer. Support vector machine (a), logistic regression (b), convolutional neural network (c) or random forest (d) can be used to establish the final multimodal fusion model.

Pathology is morphology-based, but the classification and assessment of disease is not limited to morphology, and requires reference to immunological, molecular, and clinical characteristics of patients. Based on the genome, Wang (65) mined prognostic features in H&E histopathological images of triple negative breast cancer (TNBC), and selected 48 pairs of significantly correlated image features and gene clusters through the TNBC genome map and H&E images of 44 cases, among which 4 pairs were significantly correlated with prognosis. Basavanhally (66) showed that H&E morphological characteristics and IHC molecular characteristics can replace expensive Oncotype DX risk assessment for the invasiveness of ER negative breast cancer. Yuan (67) proposed a mathematical statistical model to evaluate the proportion of lymphocytes in TNBC tumors, and the results showed that lymphocytes were related to the survival of TNBC, and the image-based evaluation results were similar to the results of gene expression spectrum detection. According to the prognostic model theory of Steyerberg (61), we can further utilize the results of image characteristics and molecular characteristics, and construct a prediction model by integrating complementary prognostic factors, which can be used to comprehensively and accurately predict the prognosis of breast cancer. Currently, integrating information from different dimensions to construct multimodal fusion models for predicting cancer biomarkers or prognosis of patients have been studied in several laboratories. The main process of building multimodal fusion models is shown in **Figure 2**. Making full use of multidimensional information for fusion modeling is of great help to improve the prediction accuracy, which will also be a direction of the development of digital pathology. Chen et al. used CNNs and GCNs to extract morphological features from digital histology images and SNNs to extract genomic signatures (68). Then they employed the Kronecker Product and a gating-based attention mechanism to fuse these deep features and further validated the approach on glioma and clear cell renal cell carcinoma (CCRCC) data from TCGA. Mobadersany et al. presented a novel method to predict outcomes of patients from histopathological images and proved that the accuracy was comparable to the traditional manual histological grading. To further improve performance, they combined histopathological images and genomic data to develop a comprehensive model called GSCNN. And its performance was significantly better than that of SCNN model and WHO paradigm based on genomic subtype and histological grading (69).

## Limitations and Future Work

Cancer histology contains rich phenotypic information and can reflect underlying molecular mechanisms and disease progression. A large number of studies have shown that deep learning of digital pathological images of tumor tissue samples can be used for cancer diagnosis, classification, drug efficacy evaluation and prognosis prediction. This method has the advantage of fast and low cost. In this work, we summarized the overall process and key steps of processing full-scan section images to help people choose better and more appropriate medical image processing methods when predicting tumor biomarkers.

However, the application of artificial intelligence (AI) technology in precision medicine has some limitations currently. Firstly, the diagnosis process of deep learning model is fuzzy and the interpretability is limited, and the lack of interpretability is unacceptable to the Medical Association (70–72). So this problem is an important obstacle to its verification and application in clinical practice. Heat map analysis provides an in-depth understanding of the histological patterns related to the prediction target, which is helpful for the interpretation of the deep learning model. Chen et al. had used this method to locate and interpret features in the study of multimodal fusion for predicting survival outcome of cancer patients (68). It can also be used as a practical tool to lead pathologists to discover the tissue regions related to biomarkers. For example, the presence of edema in glioma was not previously considered as an adverse marker by pathologists, but was detected as a recognition feature in the model of predicting cancer prognosis (69). Associated with this finding, the degree of edema may be correlated to the growth rate of cancer in previous study (73). Cao et al. verified the reliability of the deep learning model in two independent cohorts when predicting MSI with pathological images, and explained the interpretability of the model by exploring the correlation between pathological features and multi-omics signatures. This is also a method to promote clinicians to accept the application of AI in digital pathological images (74). It can be predicted that improving the interpretability of the model or establishing interpretable machine learning methods will be an important topic to be explored in the future.

Secondly, a substantive problem limiting its clinical application is the frequent workflow switching due to the limited integration of computer-aided pathological diagnosis in the current pathological workflow (70–72). Currently, the research on diagnosis and subtyping of cancer through digital pathological images is relatively mature. Some latest studies on predicting cancer prognosis, treatment response and disease progress monitoring through pathological images have been reported. Kather et al. developed a deep learning model that can directly predict microsatellite instability from H&E histological images of stomach and colorectal cancer and the AUC values ranged from 0.69 to 0.84 in independent validation datasets (17). Cao et al. explored an EPLA model with AUC of 0.8504 (95% CI: 0.7591-0.9323) in the external validation set (74). However, more histological images of patients are needed to optimize the model and improve accuracy. If a complete pathological diagnosis and prediction process through extensive analysis of various data can be established and verified clinically, it will contribute to the application of AI in precision medicine (71, 75).

Thirdly, it is difficult to unify the staining and imaging process of tissue section in different laboratories, which leads to a large number of variables in pathological images and further makes it difficult to establish models with high stability and good generalization performance. Just as molecular diagnosis relies on qualified samples and sequencing data, digital image analysis also requires strict control of sample quality, clear quality requirements for input files, and adequate training for pathologists. These requirements of digital pathological image analysis will also drive to improve the volume and accuracy of histomorphological evaluation. On the other hand, in order to promote clinical transformation, a roadmap and regulatory framework for the routine use of AI in pathology have been published (76).

Other literatures also list possible practical problems: slow implementation time of computer-aided pathology, insufficient clinical validation of computer-aided pathology, and limited impact on health economics (9, 71). The ability to overcome these limitations will determine the future of digital pathology.

## Author Contributions

GT and XShi designed the project. XX, XW, and XShi searched literatures and wrote the manuscript. YL, JY, YW, LL, XSun, PB, and BH revised the manuscript. All authors have approved the final version of the manuscript.

## Funding

This study was supported by Natural Science Foundation of Hunan, China (Grant No. 2018JJ3570), Major Project for New Generation of AI (Grant No. 2018AAA0100400), the National Natural Science Foundation of Hunan (Grant Nos. 2018JJ2098), the National Natural Science Foundation of China (Grant No. 11571052, 11731012).

## Conflict of Interest

Author XShi, GT, YL, JY and YW were employed by the company Geneis Beijing Co., Ltd. Author LL was employed by the company Beijing Shanghe Jiye Biotech Co., Ltd.

The remaining authors declare that the research was conducted in the absence of any commercial or financial relationships that could be construed as a potential conflict of interest.

## Publisher’s Note

All claims expressed in this article are solely those of the authors and do not necessarily represent those of their affiliated organizations, or those of the publisher, the editors and the reviewers. Any product that may be evaluated in this article, or claim that may be made by its manufacturer, is not guaranteed or endorsed by the publisher.
